# Cardiocerebrovascular benefits of early rhythm control in patients with atrial fibrillation detected after stroke: a systematic review and meta-analysis

**DOI:** 10.3389/fcvm.2024.1391534

**Published:** 2024-05-16

**Authors:** Liang Ma, Baofu Wang, Jiasai Fan, Hufang Zhou, Jingen Li, Weisheng Li, Xiangying Zheng, Xian Wang

**Affiliations:** ^1^ Department of Cardiology, Dongzhimen Hospital, Beijing University of Chinese Medicine, Beijing, China; ^2^ Department of Integrative Medicine Cardiology, China-Japan Friendship Hospital, Beijing, China; ^3^ Department of Cardiovascular Medicine, Mayo Clinic, Rochester, MN, United States

**Keywords:** early rhythm control, atrial fibrillation, stroke, cardiocerebrovascular events, recurrent stroke, all-cause mortality

## Abstract

**Objective:**

This study aimed to evaluate the impact of early rhythm control (ERC) on the occurrence of cardiocerebrovascular events in patients diagnosed with atrial fibrillation detected after stroke (AFDAS).

**Methods:**

A systematic search was conducted across nine databases from inception to October 15, 2023 to identify clinical trials comparing ERC with usual care interventions in AFDAS patients. The primary outcome assessed was recurrent stroke, with secondary outcomes including all-cause mortality, adverse events related to arrhythmias, and dementia.

**Results:**

Analysis of five studies, consisting of two randomized clinical trials (RCTs) involving 490 patients and three cohort studies involving 95,019 patients, revealed a reduced rate of recurrent stroke [odds ratio (OR) = 0.30, 95% confidence interval (CI) 0.11–0.80, *P* = 0.016 in RCTs; OR = 0.64, 95% CI 0.61–0.68, *P* < 0.00001 in cohort studies] and all-cause mortality (hazards ratio = 0.94, 95% CI 0.90–0.98, *P* = 0.005 in cohort studies) in the ERC group compared to the usual care group. In addition, ERC was associated with superior outcomes in terms of dementia.

**Conclusions:**

Patients with AFDAS who underwent ERC treatment exhibited a decreased risk of cardiocerebrovascular events compared to those receiving usual care. These results support the potential benefits of implementing an ERC strategy for this specific patient population.

**Systematic Review Registration:**

https://www.crd.york.ac.uk/PROSPERO/, Identifier [CRD42023465994].

## 1 Introduction

Atrial fibrillation (AF) is characterized by uncoordinated electrical conduction in the atria, leading to uncoordinated atrial systole and diastole. The estimated global prevalence is over 37 million (1). AF can result in a range of cardiocerebrovascular diseases, including stroke, heart failure, dementia, and ultimately death. Notably, AF significantly increases the risk of stroke by three to five times, highlighting its importance as a key risk factor (2). Conversely, stroke can also trigger the detection of AF, with approximately 1.5 million stroke survivors worldwide later being diagnosed with AF (3). Bernstein et al. discovered that the rate of detecting AF in patients with a history of stroke is 21.7% within a 3-year period (4). Nevertheless, there is a noticeable absence of standardized treatment guidelines for patients with AFDAS (5).

Current therapeutic approaches for AFDAS involve anticoagulant medications to reduce the risk of cardiovascular and cerebrovascular complications. To manage newly diagnosed AF, it is recommended that rate control treatment be prioritized in cases where symptoms manifest. If this approach does not provide any relief, antiarrhythmic drugs (AADs) and ablation procedures must be considered (5). However, recent research suggests that oral anticoagulation therapy in AFDAS may not significantly reduce the risk of stroke or systemic arterial embolism (6, 7). Sposato et al. found that the burden of AF in AFDAS was more severe compared to AF alone (3). Due to post-stroke neurogenic mechanisms, stroke-induced heart injury, autonomic dysfunction, and inflammatory responses, individuals with AFDAS often have a higher burden of AF, increasing the risk of recurrent stroke and exacerbating cardiovascular and cerebrovascular diseases (8, 9). Therefore, in addition to anticoagulation therapy, AFDAS patients must explore proactive antiarrhythmic treatment strategies to improve their prognosis.

The 2020 EAST-AFNET 4 trial revealed that early rhythm control (ERC) for AF patients leads to better long-term outcomes compared to usual care (UC), in which patients were primarily treated with rate control without rhythm control (10). There were no significant differences in adverse effects between the two approaches (10). However, the clinical benefit of rhythm control therapy for AFDAS patients is still uncertain. Due to the associated risks of stroke, thromboembolism, and atrio-esophageal fistula following catheter ablation (11, 12), there is a tendency in clinical practice to avoid prescribing rhythm control therapy for AFDAS considering the real clinical benefit and cost-effectiveness ratio (13).

Therefore, this study aims to investigate the impact of strengthened rhythm control in the early stages of AFDAS on cardiovascular and cerebrovascular outcomes, providing valuable evidence on the effectiveness of ERC for AFDAS.

## 2 Methods

### 2.1 Registration

This review adhered to the reporting guidelines for systematic reviews and was registered with PROSPERO, an international prospective register of systematic reviews (CRD42023465994) (14).

### 2.2 Search strategy

To search for relevant articles, we conducted search in nine databases, namely, PubMed, Embase, Cochrane Library, Web of Science, Clinical Key, ClinicalTrials.gov, CNKI, Wan Fang, and VIP databases, up to October 15, 2023 for relevant clinical trials investigating rhythm control in stroke-complicating AF. The search strategy, detailed in Supplementary Material, was based on combinations of keywords such as “stroke,” “atrial fibrillation,” “rhythm control,” and their synonyms.

### 2.3 Inclusion and exclusion criteria

The inclusion criteria for the study literature included the following: (1) subjects with a history of stroke and newly diagnosed with AF; (2) the experimental group receiving early AF diagnosis (within 1 year) received antiarrhythmic therapy, whereas the control group received usual treatment; (3) studies reporting adverse events related to cardiovascular and cerebrovascular diseases, including recurrent stroke, all-cause mortality, adverse events related to arrhythmias, and dementia; and (4) unrestricted by age, gender, race, etc.

The exclusion criteria were as follows: (1) non-clinical and irrelevant studies; (2) studies with unpublished or missing data from registered clinical trial results; (3) in case of studies with repetitive or similar data sources, retaining the most comprehensive and relevant report or literature; and (4) clinical trial protocols, reviews, meta-analyses, case reports, and conference abstracts.

### 2.4 Literature screening and data extraction

Duplicate literature was excluded, and two researchers independently conducted a preliminary review based on titles and abstracts, followed by a full-text review. Relevant information on study design and outcomes was extracted for final inclusion in this study. Two researchers independently performed data extraction, including first author, publication date, sample size, gender, follow-up period, interventions, and outcome indicators. Disagreements were resolved by discussion or, if necessary, with the assistance of a third researcher. The two researchers then independently conducted a full-text assessment to confirm the key information of the included studies. Finally, the extracted information was cross-checked by both researchers, and any discrepancies were resolved through consultation with a third researcher.

### 2.5 Quality assessment

Two quality-control reviewers assessed the quality of the literature using the Cochrane Handbook's quality assessment criteria for randomized clinical trials (RCTs) and the Newcastle–Ottawa Scale (NOS) for non-randomized controlled trials. Both quality-control officers independently assessed the quality and the risk of bias of each study. In case of a disagreement, a third reviewer performed a reassessment.

### 2.6 Outcomes and definitions

The primary outcome of this study was recurrent stroke. The secondary outcomes included all-cause mortality, adverse events related to arrhythmias, and dementia. Recurrent stroke was defined as incident stroke during follow-up. Incident stroke was determined by the primary diagnosis during admission using ICD-10-CM codes.

### 2.7 Statistical methods

Data analysis was performed using Stata14.0. Considering the limited number of included studies, a fixed-effects model was employed (15). Odds ratios (ORs) were used to analyze binary variables, while hazards ratios (HRs) were used for all-cause mortality. *P* < 0.05 was considered statistically significant.

## 3 Results

### 3.1 Study selection

**Figure 1:**
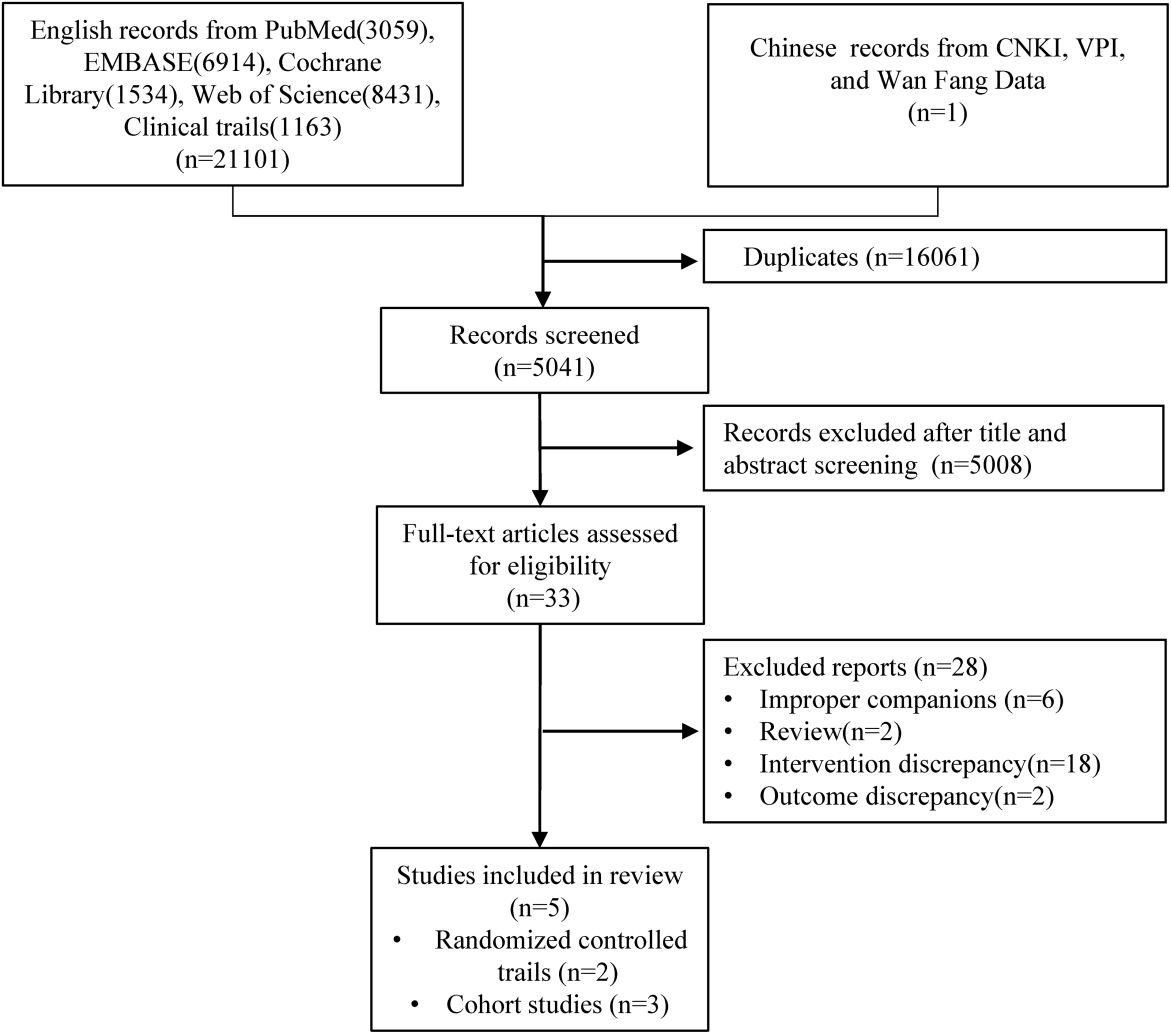
Flowchart of article selection.

Using a computer system to search the nine databases mentioned above, a comprehensive compilation of 21,102 pertinent pieces of studies on ERC for AFDAS has been amassed as of October 15, 2023. A total of 16,061 duplications were excluded from consideration. A subsequent scrutiny of titles and abstracts led to the exclusion of 5,008 articles that failed to meet the stipulated criteria. Finally, after an exhaustive review of the entire article, five studies were included for the purpose of meta-analysis and systematic review (Figure 1).

### 3.2 Basic information of studies

Five eligible studies (16–20) were published in English, involving 95,509 participants, with 22,928 in the ERC group and 7,581 in the UC group. This included two RCTs (18, 19) with 490 patients and three cohort studies (16, 17, 20) with 95,019 patients. Four studies reported recurrent stroke and mortality rates (17–20), while only one study reported dementia (16). The longest follow-up time for each study was taken as the outcome information collection point for research.

For the implementation of treatment strategies, the ERC group received either rhythm control surgery or drug treatment within 1 year of AF diagnosis. Noteworthy distinctions emerged in the approach to the UC group across studies. In their investigation, Lee et al. (16, 17) refrained from administering rhythm control therapy to patients during the observation period, whereas Sagris et al. (20) and Jensen et al. (18) initially applied rate control therapy to the UC group. If adequate rate control failed to restore the sinus rhythm (SR) or provide symptomatic relief, antiarrhythmic control therapy was introduced. In the work of Park et al. (19), usual care involved incorporating antiarrhythmic therapy 2 months after enrollment, depending on the patient's specific condition. The foundational data across all investigations depicted a semblance of comparability, with patients having an average age range of 68–75 years. Table 1 summarizes the basic characteristics reported in each study.

**Table 1:** Basic characteristics of the included studies.

Include studies (years)	Study design	Sample size		Male (%)		Age, mean (SD, years)		First episode AF, *n* (%)		Paroxysmal AF, *n* (%)		Persistent AF, *n* (%)		CHA2-DS2-VASc score		Anticoagulants (NOAC/VKA)		Antiplatelets		Intervention		Max follow-up (years)	Outcomes
		T	C	T	C	T	C	T	C	T	C	T	C	T	C	T	C	T	C	T	C		
Park et al. (19)	RCT	178	95	60.7	64.2	68.9 (8.9)	70.1 (7.8)	NR	NR	94 (52.8)	48 (50.5)	84 (47.2)	47 (49.5)	4.4 (1.5)	4.3 (1.6)	166 (93.3)	89 (93.7)	61 (34.3)	34 (35.8)	ERC	UC	1	1. Recurrent stroke 2. Mortality 3. Adverse events related to arrhythmia
Jensen et al. (18)	RCT	110	107	41	47	71 (7.4)	72 (7.8)	32 (29.1)	41 (38.3)	49 (44.5)	36 (33.6)	29 (26.4)	30(28.0)	5.0 (1.5)	5.0 (1.5)	106 (96.4)	92 (86.0)	17 (15.5)	29 (27.1)	ERC	UC	8	1. Recurrent stroke 2. Mortality 3. Adverse events related to arrhythmia
Sagris et al. (20)	Cohort study	143	87	51.7	56.3	74 (8.9)	75.3 (8.1)	NR	NR	143 (100)	87 (100)	NR	NR	3.4 (1.5)	3.4 (1.5)	33 (40.2)	37 (45.1)	48 (35.6)	33 (40.2)	ERC	UC	10	1. Recurrent stroke 2. Mortality
Lee et al. (17)	Cohort study	12,284	41,135	52.9	53	72.2 (9.6)	72 (10.9)	NR	NR	NR	NR	NR	NR	5.5 (1.6)	5.5 (1.6)	12,284 (100)	41,135 (100)	NR	NR	ERC	UC	8	1. Recurrent stroke 2. Mortality
Lee et al. (16)	Cohort study	10,213	31,157	57		70 (11)		NR	NR	NR	NR	NR	NR	NR	NR	NR	NR	NR	NR	ERC	UC	8	1. All dementia 2. Alzheimer dementia 3. Vascular dementia

Values are *n* (%); NR, not reported or cannot combine; SD, standard deviation; T, treatment; C, control; RCT, randomized clinical trial; ERC, early rhythm control, including AADs and catheter ablation; UC, usual care; NOAC, non-vitamin K oral anticoagulant; VKA, vitamin K antagonist.

### 3.3 Risk of bias

In two RCTs (18, 19), the study by Jensen et al. (18) randomized participants using a central randomization list and blinded the outcomes. Park et al. (19) did not provide a detailed description of the random allocation method, and one subject was excluded from both the experimental and control groups. Due to the use of rhythm control methods, such as radiofrequency ablation in both studies, blinding of subjects was not possible, but the impact on outcome indicator detection was not significant. Tables 2 and 3 provide additional information.

**Table 2:** Risk of bias assessment of included randomized controlled trials.

Study	Random sequence generation	Allocation concealment	Blinding of participants and personnel	Blinding of outcome assessment	Free of infrequent missing outcome data	Free of selective reporting	Other bias
Park et al. (19)	Probably yes^a^	Definitely no Open-label	Definitely no Open-label	Definitely yes^b^	Definitely yes^c^	Probably yes^d^	Probably yes^d^
Jensen et al. (18)	Definitely yes^e^	Definitely no Open-label	Definitely no Open-label	Definitely yes Blinded outcome assessment	Definitely yes^d^	Probably yes^d^	Probably yes^d^

^a^ Method for generating a randomization sequence was not clearly reported. We judged that randomization sequence generation was likely not achieved given that it used a one-to-one basis according to instructions.

^b^ The blind method was not used, but the outcome indicators were not affected.

^c^ No more than patients assigned to early rhythm control had no information about the history of stroke and were excluded from this subgroup analysis, but it is unlikely to affect the outcomes.

^d^ Generally balanced baseline characteristics across groups.

^e^ Randomization was performed by an independent statistician using a central randomization list.

**Table 3:** Risk of bias assessment of included cohort studies.

	Selection				Comparability	Outcome		
Study	Representativeness of the cohort	Selection of the non-exposed cohort	Ascertainment of exposure	Demonstration that outcome of interest was not present at the start of the study	Comparability of cohorts on the basis of design or analysis time to follow-up	Assessment of outcome	Follow-up long enough for outcomes to occur	Adequacy of follow-up cohorts
Lee et al. (17)	1	1	1	1	2	1	1	1
Sagris et al. (20)	1	0^a^	1	1	1^b^	1	1	0^c^
Lee et al. (16)	1	1	0^d^	1	2	0^d^	1	0^d^

^a^ The patients in the control group all have AF that spontaneously converts to sinus rhythm.

^b^ The baseline characteristic of the coronary history was different (*P* < 0.05).

^c^ Forty-five patients (15.2%) were lost to the scheduled follow-up (from 6 to 75 months).

^d^ Not mentioned.

Three cohort studies (16, 17, 20) were prospective. For both the studies of Lee et al. (17) and Sagris et al. (20), relevant medical records were extracted. In the study of Sagris et al. (20), the control group consisted of patients with AF that could spontaneously revert to sinus rhythm, while the experimental group consisted of patients who could revert to sinus rhythm after AF treatment, consequently affecting the intergroup comparability. At the same time, 15.2% of the patients dropped out, which may affect the outcome indicators. Therefore, no score was given for the relevant items. Table 3 displays more detailed items in each domain. The NOS scores of the three studies were 9 (17), 6 (20), and 6 (16), respectively.

### 3.4 Primary outcome

#### 3.4.1 Recurrent stroke

**Figure 2:**
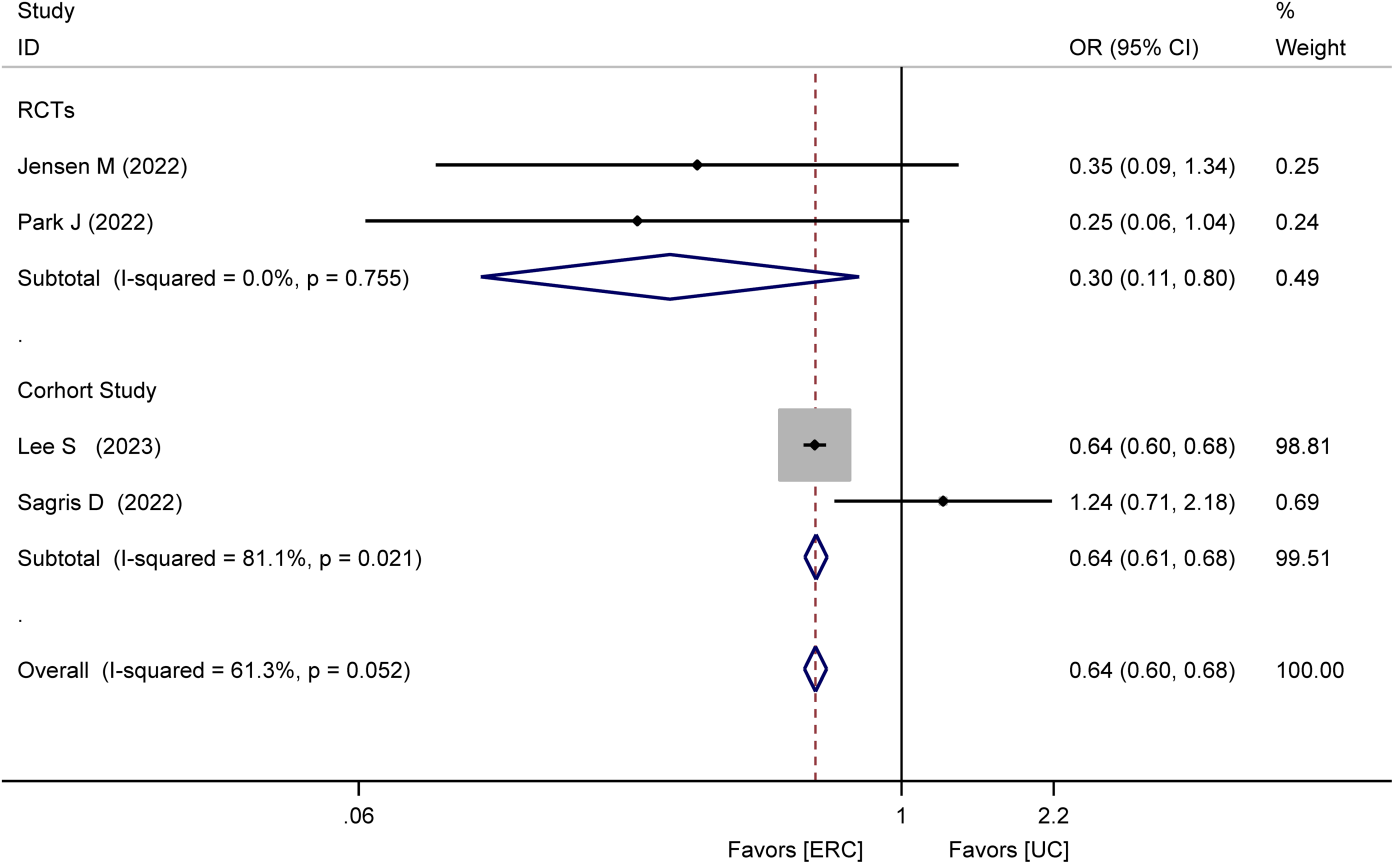
Forest plot of meta-analysis of RCTs and cohort studies involving recurrent stroke between ERC vs. UC.

Four studies (17–20), comprising two RCTs (18, 19) and two cohort studies (17, 20), assessed the incidence of recurrent stroke. The meta-analysis found that the recurrence stroke was lower in the ERC group than in the UC group (OR = 0.64; 95% CI 0.60–0.68; *P* < 0.00001; Figure 2). In the RCTs, the meta-analysis showed a statistically significant reduction in the occurrence of recurrent strokes in the ERC group than in the UC group after treatment (OR = 0.30, 95% CI 0.11–0.80, *Z* = 2.40, *P* = 0.02). In the cohort study, the meta-analysis showed a statistically significant lower incidence of recurrent stroke events in the ERC group than in the UC group (OR = 0.64, 95% CI 0.61–0.68, *Z* = 15.05, *P* < 0.00001).

### 3.5 Secondary outcome

#### 3.5.1 All-cause mortality rate

**Figure 3:**
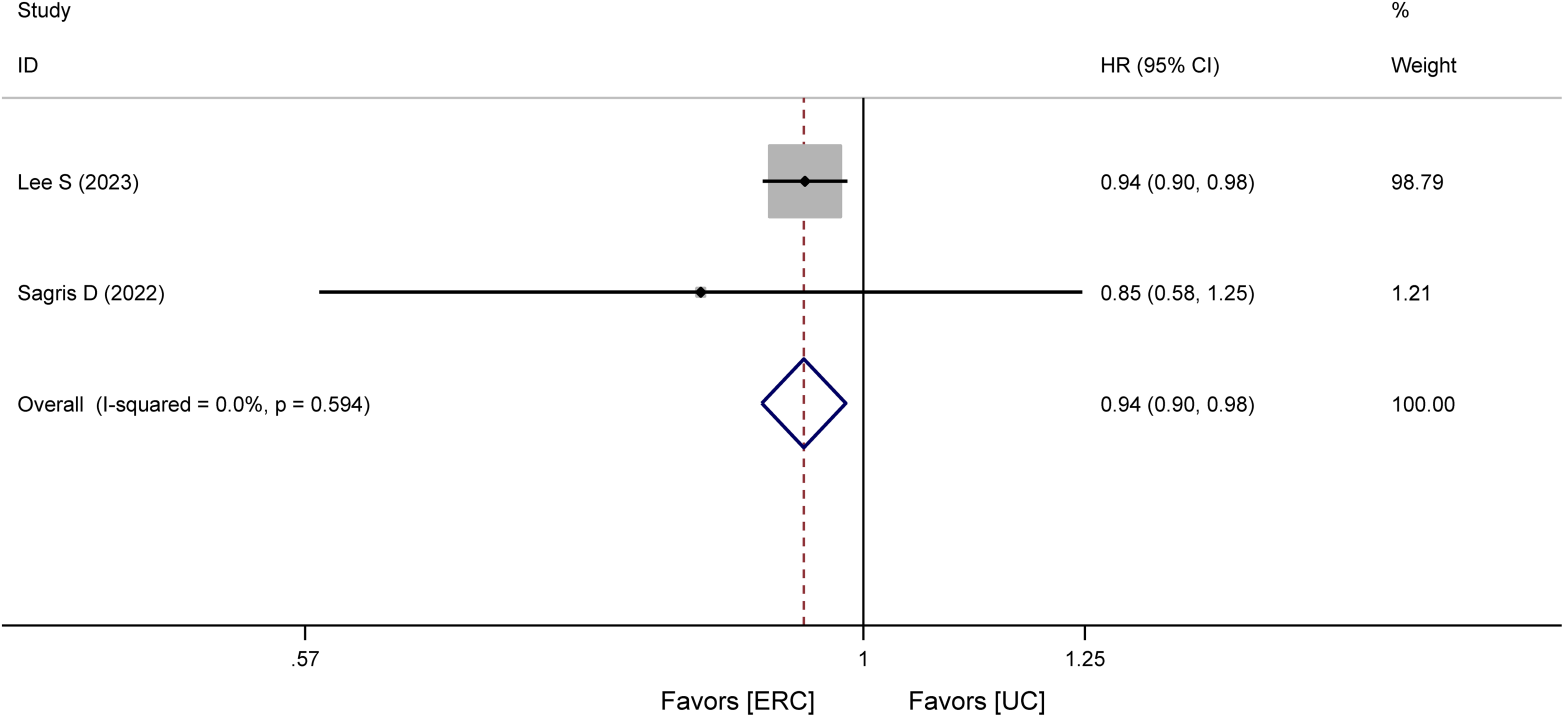
Forest plot of meta-analysis of cohort studies involving all-cause mortality between ERC vs. UC.

Three studies (17, 18, 20), including one RCT (18) and two cohort studies (17, 20), analyzed all-cause mortality. In the RCT (18), all-cause mortality within 8 years of treatment was lower in the ERC group [11% (10%)] than in the UC group [21% (20%)]. In cohort studies, the HR for all-cause mortality was statistically significant, that is, HR = 0.94, 95% CI 0.90–0.98, *P* = 0.005, as shown in Figure 3. This illustrates a significantly reduced risk of all-cause mortality in the ERC group.

#### 3.5.2 Adverse events related to arrhythmia

Regarding the safety aspects of rhythm control treatment, one study (18) reported adverse events associated with rhythm control therapy. The results showed a higher incidence of adverse events in patients receiving ERC than in to those receiving UC [3 (3%) vs. 2 (2%)]. These events included drug-induced bradycardia, syncope attributed to rhythm control therapy, and implantation of a pacemaker or other cardiac devices in the ERC group. In the UC group, there were two events related to hospitalization for AF and drug toxicity of AF-related drug therapy.

Moreover, another study (19) reported arrhythmia-related events. The results showed that there was no statistically significant difference in the incidence of ERC compared to UC [5 (2.8%) vs. 1 (1.1%); *P* = 0.372]. These adverse arrhythmia events included pacemaker implantations (permanent or temporary) in patients with potential sinus disease (four cases in total) and a single case of syncope.

#### 3.5.3 Dementia

One cohort study (16) reported the incidence of dementia in AFDAS patients. The result showed that ERC treatment was superior to UC treatment in reducing the incidence of dementia, Alzheimer's disease, and vascular dementia (HR: 0.27 vs. 0.33; 0.05 vs. 0.06; 0.21 vs. 0.26).

## 4 Discussion

This systematic review and meta-analysis revealed that ERC may decrease the risk of recurrent stroke in patients with AFDAS. In addition, ERC was also associated with a lower risk of all-cause mortality and dementia. This study represents the initial systematic review and meta-analysis investigating the effectiveness and safety of ERC in patients with AFDAS.

This study included five studies with a total of 95,509 participants, comparing ERC with UC for patients with AFDAS in relation to cardiocerebrovascular events. The findings indicate that ERC can reduce the risk of recurrent stroke and the incidence of all-cause mortality and alleviate the occurrence of dementia. There was no significant difference in arrhythmia-related adverse event rates between the two groups.

Recent studies have shown that AFDAS can be categorized into neurogenic atrial fibrillation (NAF) and cardiogenic atrial fibrillation (CAF), each with distinct pathological mechanisms that contribute to the occurrence of atrial fibrillation (8, 21). Sposato et al. (7) found that, after excluding the influence of confounding factors such as oral anticoagulants, when comparing patients with AFDAS, stroke with SR, and AF known before the stroke (KAF), there was no statistically significant difference in the 1-year ischemic stroke recurrence rates between AFDAS and SR. Interestingly, the highest incidence of recurrent stroke was observed in KAF. This indicated that the timing of AF onset in stroke patients may be influenced by different pathophysiological factors, including cardiogenic and neurogenic origins.

NAF may result from damage to the dorsal anterior insular cortex, which regulates the parasympathetic nerve after ischemic stroke. This damage can cause an imbalance in the heart and the autonomic nervous system, leading to an upregulated sympathetic nerve activity and a subsequent myocardial injury (22, 23). Compared to SR, no difference was found in stroke incidence; therefore, for NAF, the antiarrhythmic therapy can be appropriately relaxed. However, it is more worthy to monitor changes in heart function as the incidence of acute heart failure or worsening heart failure is 3.6 times compared to KAF (6). ByIn contrast, CAF in AFDAS may stem from cardiac pathology, a well-known high-risk factor for stroke. CAF could potentially precede stroke onset but remain undetected due to the technical limitations of ECG detection. The duration of AF has been identified as a significant factor influencing the risk of stroke (24). AFDAS patients, especially those undetected with AF before the stroke, may have longer AF duration, leading to a higher incidence of stroke and adverse events related to cardiovascular and cerebrovascular events. Therefore, effective antiarrhythmic control therapy, such as the use of ERC, is necessary for this population.

ERC not only alleviates the symptoms of AF but also reduces the atrial fibrillation burden and the hospitalization time (10). Its positive impact extends to cardiac structural and functional recuperation, promoting hemodynamic stability, preventing thrombosis, reducing recurrent stroke rates and adverse events, and enhancing cardiac output and cerebral blood perfusion. These benefits are crucial for protecting the cognitive function (25). Although there is currently a lack of controlled studies comparing NAF and CAF, we believe that ERC for AFDAS may provide greater clinical benefits for patients, regardless of whether they have NAF or CAF, given the potential stroke prevention and cardiac function preservation.

The impact of stroke duration on decision-making for ERC treatment in AFDAS remains unexplored. Regrettably, our study lacked sufficient data for subgroup analysis, limiting our ability to make definitive conclusions. However, Song et al. found that performing radiofrequency ablation surgery on AF patients within 3 months of stroke resulted in similar postoperative complication rates as those without a stroke history. Moreover, there was a reduced risk of recurrent stroke after 12 months of follow-up compared to usual care (26). This evidence suggests that ERC could be a safe and effective treatment for AF patients who recently suffered a stroke within 3 months.

Regarding specific rhythm control treatment choices, no study is currently analyzing the difference between AADs and radiofrequency ablation in patients with AFDAS. Although ablation tools and procedures have advanced, radiofrequency ablation may still carry a risk of subclinical or clinical thromboembolism with an estimated stroke risk of 0.5%–1% during the procedure (27). AADs also have drawbacks, including adverse reactions like arrhythmia and QT interval prolongation, and often require personalized dose adjustments and dynamic monitoring. As direct comparison is lacking, clinicians typically make treatment decisions based on individual patient's characteristics, such as age, heart function, stroke history, and other complications. In some cases, a combination of both AADs and radiofrequency ablation may be necessary to achieve optimal rhythm control.

ERC can decrease cerebrovascular incidence in AFDAS patients and benefit other frail patients affected by AF. Currently, catheter ablation is the first choice in ERC treatment. La Fazia et al. (28) found that human immunodeficiency virus (HIV) patients with AF have more serious inflammation and oxidative stress caused by HIV infection, which can contribute to atrial remodeling and AF burden (28). For HIV + AD patients, undergoing catheter ablation earlier can significantly reduce the incidence of atrial arrhythmias. Sohns et al. (29) found that for end-stage HF with AF, CA was associated with a lower rate of all-cause mortality.

### 4.1 Comparison with previous studies

This study is the first systematic review and meta-analysis to examine the treatment benefits of ERC in patients with AFDAS. The investigation used a meticulous approach and conducted multiple searches across various online platforms to ensure a comprehensive and exhaustive search strategy. The quality assessment was reasonable, and the included literature exhibited a high level of homogeneity.

### 4.2 Limitation

Numerous constraints necessitate careful consideration when interpreting our findings. First, the scope of included studies was limited, preventing us from conducting meta-analyses for AF-related subtypes and other adverse events. While we performed a comprehensive literature search for all eligible trials, this limitation may introduce some bias into our study. Furthermore, potential biases may have arisen in the timing of rhythm control treatments within the UC group. It is also possible that some patients with ischemic stroke and KAF had undiagnosed atrial fibrillation in their medical history. Moreover, the complexities of ECG presented technical challenges, making it difficult to determine whether arrhythmias in stroke-complicated AF patients are of cardiac or neurogenic origin.

## 5 Conclusion

The use of ERC in patients with AFDAS has shown superior outcomes compared to usual care in terms of reducing the risk of cardiocerebrovascular events. However, considering the limited number of studies included in our analysis, the results of this study need to be further validated.

## Data Availability

The original contributions presented in the study are included in the article/**Supplementary Material**; further inquiries can be directed to the corresponding author.
