# Supplementary material for: Cardiocerebrovascular benefits of early rhythm control in patients with atrial fibrillation detected after stroke: a systematic review and meta-analysis
Source: Front Cardiovasc Med. 2024 May 16;11:1391534. doi: 10.3389/fcvm.2024.1391534 (PMC11137666; doi:10.3389/fcvm.2024.1391534)
Supplement: Supplementary file 1 [file Table1.docx]

**Cardiocerebrovascular benefits of early rhythm control in patients with atrial fibrillation detected after stroke: a systematic review and meta-analysis**

Supplementary materials

## Search strategies:

1. Stroke [MeSH]
2. Stroke[Ti/Ab] OR Strokes[Ti/Ab] OR Cerebrovascular Accident[Ti/Ab] OR Cerebrovascular Accidents[Ti/Ab] OR CVA[Ti/Ab] OR CVAs [Ti/Ab] OR Cerebrovascular Apoplexy[Ti/Ab] OR Apoplexy, Cerebrovascular[Ti/Ab] OR Vascular Accident, Brain[Ti/Ab] OR Brain Vascular Accident[Ti/Ab] OR Brain Vascular Accidents[Ti/Ab] OR Vascular Accidents, Brain[Ti/Ab] OR Cerebrovascular Stroke[Ti/Ab] OR Cerebrovascular Strokes[Ti/Ab] OR Stroke, Cerebrovascular[Ti/Ab] OR Strokes, Cerebrovascular[Ti/Ab] OR Apoplexy[Ti/Ab] OR Cerebral Stroke[Ti/Ab] OR Cerebral Strokes[Ti/Ab] OR Stroke, Cerebral[Ti/Ab] OR Strokes, Cerebral[Ti/Ab] OR Stroke, Acute[Ti/Ab] OR Acute Stroke[Ti/Ab] OR Acute Strokes[Ti/Ab] OR Strokes, Acute[Ti/Ab] OR Cerebrovascular Accident, Acute[Ti/Ab] OR Acute Cerebrovascular Accident[Ti/Ab] OR Acute Cerebrovascular Accidents[Ti/Ab] OR Cerebrovascular Accidents, Acute[Ti/Ab]
3. 1 or 2
4. Atrial Fibrillation [MeSH]
5. Atrial Fibrillation[Ti/Ab] OR Atrial Fibrillations[Ti/Ab] OR Fibrillation, Atrial[Ti/Ab] OR Fibrillations, Atrial[Ti/Ab] OR Auricular Fibrillation[Ti/Ab] OR Auricular Fibrillations[Ti/Ab] OR Fibrillation, Auricular[Ti/Ab] OR Fibrillations, Auricular[Ti/Ab] OR Persistent Atrial Fibrillation[Ti/Ab] OR Atrial Fibrillation, Persistent[Ti/Ab] OR Atrial Fibrillations, Persistent[Ti/Ab] OR Fibrillation, Persistent Atrial[Ti/Ab] OR Fibrillations, Persistent Atrial[Ti/Ab] OR Persistent Atrial Fibrillations[Ti/Ab] OR Familial Atrial Fibrillation[Ti/Ab] OR Atrial Fibrillation, Familial[Ti/Ab] OR Atrial Fibrillations, Familial[Ti/Ab] OR Familial Atrial Fibrillations[Ti/Ab] OR Fibrillation, Familial Atrial[Ti/Ab] OR Fibrillations, Familial Atrial[Ti/Ab] OR Paroxysmal Atrial Fibrillation[Ti/Ab] OR Atrial Fibrillation, Paroxysmal[Ti/Ab] OR Atrial Fibrillations, Paroxysmal[Ti/Ab] OR Fibrillation, Paroxysmal Atrial[Ti/Ab] OR Fibrillations, Paroxysmal Atrial[Ti/Ab] OR Paroxysmal Atrial Fibrillations[Ti/Ab]
6. 4 or 5
7. rhythm control[Ti/Ab] OR ablation[Ti/Ab] OR cardioversion[Ti/Ab] OR antiarrhythmic drugs[Ti/Ab] OR amiodarone[Ti/Ab] OR dronedarone[Ti/Ab] OR flecainide[Ti/Ab] OR propafenone[Ti/Ab] OR pilsicainide[Ti/Ab] OR sotalol[Ti/Ab]
8. 3 and 6 and 7
